# Public Interest in Online Information on Recurrent Urinary Tract Infections Is Greatest for Information with the Poorest Publication Quality

**DOI:** 10.3390/pathogens13121125

**Published:** 2024-12-20

**Authors:** Sapna Thaker, Justin Y. H. Chan, Karan N. Thaker, Rebecca A. Takele, Abigail F. Newlands, Kayleigh Maxwell, Yasin Bhanji, Melissa Kramer, Kymora B. Scotland

**Affiliations:** 1 Department of Urology, University of California Los Angeles, Los Angeles, CA 90095, USA; sapnathaker@g.ucla.edu (S.T.); karan.thaker@ucla.edu (K.N.T.); 2 Department of Surgery, Division of Urology, University of Toronto, Toronto, ON M5S3H2, Canada; justinyhchan@gmail.com; 3 SUNY Downstate Department of Orthopaedic Surgery and Rehabilitation Medicine, Brooklyn, NY 11225, USA; rebecca.takele@downstate.edu; 4 School of Psychology and Clinical Language Sciences, University of Reading, Reading RG6 7BE, UK; abbi@liveutifree.com; 5 Live UTI Free Ltd., D18 NW62 Dublin, Ireland; kayleigh@liveutifree.com; 6 Department of Urology, David Geffen School of Medicine, University of California Los Angeles, Los Angeles, CA 90095, USA; ybhanji@gmail.com (Y.B.); kscotland@mednet.ucla.edu (K.B.S.)

**Keywords:** urinary tract infections, recurrent urinary tract infections, patient concerns, patient engagement, health-related information

## Abstract

*Background*: Urinary tract infections (UTIs) are among the most prevalent bacterial infections. With many patients turning to the Internet as a health resource, this study seeks to understand public engagement with online resources concerning recurrent UTIs (rUTIs), assess their reliability, and identify common questions/concerns about rUTIs. *Methods*: Social media analysis tool BuzzSumo was used to calculate online engagement (likes, shares, comments, views) with information on rUTIs. The reliability of highly engaged articles was evaluated using the DISCERN questionnaire. Highly engaged categories were entered as keywords in Google Trends to quantify search interest. To categorize patient-specific concerns, a database containing anonymously collected patient questions about rUTIs was created. *Results*: BuzzSumo revealed four search categories: general information, treatment, causes, and herbal remedies. DISCERN scores indicated moderate reliability overall; however, the “herbal remedies” category demonstrated poor reliability despite high engagement. Google Trends analysis highlighted “causes” and “treatment” searches as highest in relative interest. The 10 most popular categories of concern were antibiotics, microbiome, vaccines, prevention, pelvic pain, sex, testing, symptoms, diet/lifestyle, and hormones. *Conclusions*: People living with rUTIs demonstrate key concerns and often seek information online, yet articles with high engagement often contain unreliable information. Healthcare professionals may consider counteracting misinformation by providing evidence-based information online about rUTIs.

## 1. Introduction

Urinary tract infections (UTIs) affect 50–60% of the female population and 20% of males [1,2]. Within this population, recurrent UTI (rUTI) rates of up to 54.2% and 15.7% have been documented for women and men, respectively [3]. The burden of rUTIs on individual patients and on society is significant [4]. On an individual level, rUTIs are associated with anxiety, depression, and a decreased quality of life. On a societal level, rUTIs have a significant socio-economic impact, with consultations for UTIs in the United States alone amounting to approximately USD 1.6 billion [2]. Given the prevalence of recurrent UTIs amongst the general population, it follows that this is one of the most queried subjects on the internet amongst patients looking to learn more about their condition and management recommendations.

With the advent of the Internet and rapid growth of social media, an increasing number of patients are engaging with online resources to learn more about their health. It is estimated that more than 40% of healthcare consumers are using social media for their healthcare needs [5]. Access to these online media may be beneficial to both healthcare professionals and patients. Firstly, it allows for reputable institutions and practitioners to disseminate evidence-based recommendations to the general public and increase health literacy [6]. It also allows for patients to have more autonomy in their own health by providing them with information to make informed decisions and serves as an avenue for patients to seek psychosocial support [5,7]. Despite the benefits provided by the Internet and social media, information from these sources also comes with certain risks. Previous studies have reported the prevalence of misinformation on social media platforms to be as high as 97% in certain health-related topics [6]. This misinformation can result in significant consequences, as evidenced by the COVID-19 pandemic, where misinformation increased vaccine hesitancy, lowered vaccine rates, and ultimately increased deaths [8].

Given the wide availability of information available online and the lack of verified information on rUTIs, this study aims to characterize the public’s interaction with online information on rUTIs, assess the reliability and quality of resources available on the web relating to urinary tract infections, and categorize patient concerns.

## 2. Materials and Methods

This study was performed using a variety of tools as described below.

### 2.1. BuzzSumo

The analytical platform BuzzSumo [9] was utilized to identify engagement with online information related to rUTIs between 2016 and 2021. Total engagements reported from BuzzSumo were the sum of shares on various social media platforms (Facebook, Instagram, Twitter/X, YouTube, Pinterest, and Reddit). The following search terms were employed: “recurrent urinary tract infection remedy”, “recurrent urinary tract infection supplement”, “recurrent urinary tract infection herbal”, “UTI supplement”, “UTI remedy”, and “UTI herbal”.

### 2.2. DISCERN Reliability Instrument

The top ten articles in English with the highest number of engagements, according to BuzzSumo, were evaluated using the DISCERN instrument (Supplementary Materials) [10]. DISCERN assigns reliability scores to health-related articles on a 5-point scale based on a series of 16 questions. Resources with poor reliability received scores of 1–2.9, moderate reliability received scores of 3–4, and scores higher than 4 were considered high-reliability resources. Overall reliability scores were the average of reliability scores assigned by three independent evaluators, who were experienced medical staff of our research team.

### 2.3. Google Trends

The top ten articles and videos from BuzzSumo were organized into four general categories and utilized as keywords on Google Trends [11] to evaluate public interest in top search terms related to rUTIs from 2016 to 2021. Google Trends reports relative search interest on a search volume index (SVI) scale from 0 to 100, with an SVI of 100 marking the highest relative level of interest during the five-year period analyzed.

### 2.4. Categorization of Patient Concerns

Since many online queries about rUTIs were non-specific, and to discern a more granular understanding of these concerns, we compiled an Airtable database of patient concerns based on surveys anonymously administered to individuals with rUTIs. A total of 1067 questions were analyzed. Unrelated questions and personal anecdotes from these surveys were excluded. The remaining questions (*n* = 709) were selected for further categorization.

## 3. Results

**Figure 1:**
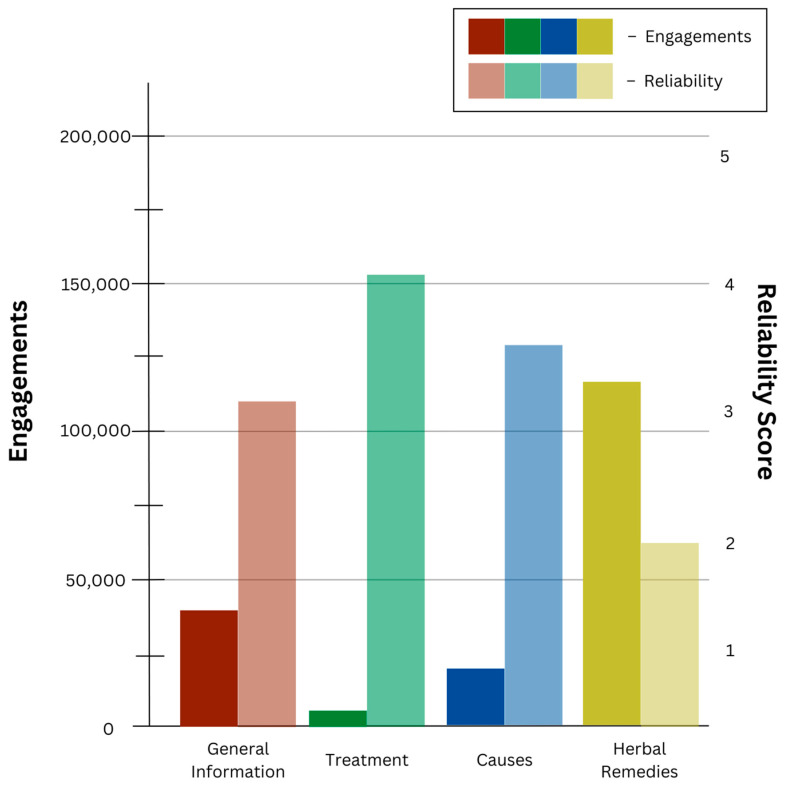
BuzzSumo social media and DISCERN reliability analysis results displaying engagements with the four general search categories of interest with their respective mean reliability scores. DISCERN reliability scores can be interpreted as follows: 1–2.9 = poor reliability; 3–4 = moderate reliability; and >4 = high reliability.

BuzzSumo was used to analyze online information related to rUTIs between August 2016 and August 2021 to evaluate engagement levels. The 40 articles with the highest engagement on popular social media platforms (Facebook, Instagram, Twitter/X, Reddit, and Pinterest) revealed the following four categories of search terms: causes, general information, herbal remedies, and treatment (Figure 1). The top 10 articles related to these four categories had total engagements (likes, shares, views, and comments) of 18,066, 31,177, 115,809, and 4060, respectively (Figure 1).

The reliability of the top ten articles with the highest engagement, according to BuzzSumo, was quantified using the DISCERN instrument. The scores from Question 16 on the DISCERN tool, indicating overall publication quality and average scores from all three reviewers, are displayed in Table 1. The articles from the “treatments” category were of high reliability, with an average score of 4.02. Articles from the “general information” and “causes” categories received mean scores of 3.21 and 3.58, respectively, indicating moderate reliability. The “herbal remedies” articles were categorized to be of poor reliability, with an average score of 2.26, despite having the highest engagements. In contrast, the “treatment” category articles had the highest reliability but the lowest level of public engagement.

**Figure 2:**
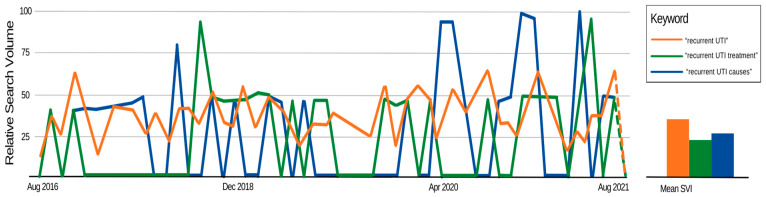
Google Trends results revealing the general recurrent UTI, recurrent UTI treatment, and recurrent UTI causes categories to be search terms of interest.

To see online interest in each of the four categories as search terms with regard to rUTIs, they were analyzed on Google Trends using the keyword approach: “[category name] recurrent UTI”, returning the results displayed in Figure 2. The general term “recurrent UTI” was found to be of high interest relative to other search terms, with a mean SVI of 38. The “treatments” and “causes” categories were also relatively high-interest search terms, with mean SVIs of 24 and 27, respectively.

Our database of patient questions (*n* = 709) revealed ten general categories of concerns: antibiotics, microbiome, UTI vaccines, prevention, pelvic pain, sex, testing, symptoms, diet/lifestyle, and hormones (Table 2). The four most popular categories of concern were UTI vaccines, symptoms, testing, and antibiotics, with 126, 103, 77, and 77 questions regarding the respective subjects. The UTI vaccine questions concerned primarily the existence of a UTI vaccine, whether UTI vaccines work against multiple organisms, and vaccine contraindications. The symptom-related questions inquired about common and uncommon symptoms of rUTI, as well as how to distinguish rUTI symptoms from other diseases. Testing and antibiotics questions had to do with testing methods and their efficacy for rUTI, which antibiotics are appropriate for rUTI, and potentially adverse impacts of long-term antibiotic use.

## 4. Discussion

In this study, we found that the categories in order from highest engagement to lowest engagement by the public as it pertains to rUTI between August 2016 and August 2021 were herbal remedies, general information, causes, and treatment. Mean SVI values for these high-engagement BuzzSumo categories varied between 24 and 38, indicating that rUTI and its related subjects are popular online search terms amongst the general population. While these categories are commonly queried online, the overall reliability of articles was moderate.

When making decisions regarding managing rUTIs, the Internet and social media serve as important sources of information for patients and act as a tool for increased self-efficacy in health. However, in our study, we found that the highest engagement categories had the lowest DISCERN reliability scores, while the lowest engagement categories had the highest DISCERN reliability scores. The category with the highest engagement within our study was “herbal remedies”, demonstrating the lowest DISCERN reliability score of 2.26. Conversely, the “treatments” category had the highest reliability with a DISCERN score of 4.02 but the lowest engagement levels. These findings may be expected since “treatments” are commonly discussed in the context of clinical consultation with a medical professional, thus requiring relatively less independent exploration by patients. However, despite the increasing interest in and popularity of complementary and alternative medicine (CAM) (which includes herbal remedies) over the last several decades in the management of genito-urinary disease, most patients do not discuss the use of CAM with their physicians [12]. In fact, it has been reported that only one-third of patients discuss the use of CAM with their physicians, with patients who are male, young adults, and/or from ethnic minority backgrounds demonstrating the lowest disclosure rates [12,13]. Several studies have also documented discrepancies between physician and patient attitudes toward CAM, with many physicians unlikely to encourage its use or discuss it [14,15,16,17]. Since it appears that many patients are hesitant to discuss these topics with their physician directly and many physicians are unlikely to raise the topic for discussion, it is unsurprising that an increasing number of patients seek out other sources of information, particularly on the Internet. Even more, this study’s findings that this highly sought-after information is of questionable reliability are particularly concerning.

Furthermore, it is not unexpected that “herbal remedies”, “treatments”, and treatment-related topics were the topics with the highest engagement online and the most queried within our own questions database for individuals with rUTIs. A focus group study performed by Scott et al. identified two predominant areas of concern from the perspective of women suffering from rUTIs. These themes of concern included (1) negative impacts of taking antibiotics for the prevention and treatment of rUTIs and (2) resentment towards the medical profession for the current management of rUTIs. The categories were derived from patients’ concerns regarding antibiotic resistance, anger towards physicians who “throw antibiotics” at them, and the belief that more research should be conducted on non-antibiotic options for prevention and treatment of UTIs [18]. These findings are further corroborated by a qualitative analysis of a post on a web forum hosted by the Cystitis and Overactive Bladder Foundation, which found that many women sought non-antibiotic treatment for their rUTIs [1]. These results highlight the importance of healthcare providers including CAM and other alternatives to antibiotics in discussions with their patients.

In our database, questions regarding a UTI vaccine were the most prevalent. The interest in a vaccine may be due to a variety of reasons. As aforementioned, concerns regarding long-term antibiotic use and resistance may be prompting patients to look for an alternative modality of treatment such as a vaccine [18]. Furthermore, in Europe, immuno-prophylaxis against UTIs has already been a formalized recommendation presented by the European Association of Urology [19]. However, in North America, although the approval of UTI vaccines is in progress, the American Urological Association and the Canadian Urological Association have not yet made similar recommendations. Rather, they have suggested the use of vaccines in managing UTIs to be an up-and-coming therapeutic [20,21]. Due to this discrepancy in recommendations, availability, and variable access to a UTI vaccine, patients who have failed antimicrobial therapy are likely anticipating such a vaccine soon and are therefore interested in learning more about it.

This study found that many of the topics related to rUTIs with the highest public engagement were also some of the least reliable resources. At best, the maximum DISCERN score assigned in our study to a category relating to rUTIs was 4.02. This indicates that commonly accessed online rUTI information has some shortcomings, false facts, or gaps in knowledge. This is worrisome as online information has been shown to have an impact on patients’ healthcare outcomes. A 2013 Pew Research study found that one in every three Americans had sought online information to make an initial diagnosis regarding any health condition. Of those, 46% self-diagnosed themselves after finding information suggesting that their condition required the attention of a medical professional. However, 18% of self-diagnosers found that their initial diagnosis made from online information was inaccurate after consulting a medical professional [22]. More broadly, this study also found that 77% of Internet users had utilized a broad search engine such as Google or Yahoo to investigate health-related information, while merely 13% of users began their research at a health information-specific platform, such as WebMD. Considering the high usage of the Internet for health-related information, it is essential for the public to understand how to critically analyze online information to assess its reliability. It is also important for policymakers to develop solutions such as fact-checking and implementing credibility labels on a regulatory level to make evidence-based information clearer to readers.

This paper provides key insights into the topics highly queried by the public regarding UTIs and the reliability of popular online sources. However, several limitations should be acknowledged. Firstly, whilst Google Trends and BuzzSumo were effective tools for the assessment of the public’s engagement with topics related to rUTIs, it is worth considering that these tools do not provide user demographic information. This limits our ability to identify the populations who most commonly use online resources to assist in managing their rUTIs and are at the highest risk of consuming misinformation. Another limitation of this study is that searches and search results may overrepresent more extreme sufferers of rUTIs. Furthermore, additional research is required to explore additional social media platforms, allowing further evaluation of a broader population of rUTI patients. For example, Snapchat and TikTok are most commonly used by people aged 18 to 29 years old, with 75% and 55% of this age group subscribing to each platform, respectively [23]. It is also within this age group that women have been found to have a high UTI incidence rate of 0.5 episodes/annum [24]. Finally, whilst Google is the leading search engine worldwide, further research specifically exploring alternative search tools such as Yahoo, Yandex, Mail.Ru, Bing, Baidu, Shenma, and Haosou would be beneficial, given that these are especially common in countries outside of North America and Europe [25].

## 5. Conclusions

Online discussions related to rUTIs receive high engagement from the general public. However, the topics with the highest engagement have the poorest reliability scores. Vaccines for rUTIs are of high interest amongst individuals searching for information online. Healthcare providers may benefit patients by recommending and contributing to reliable online sources of rUTI information.

## Figures and Tables

**Table 1:** Table of DISCERN scores for Question 16 (“Based on the answers to all of the above questions, rate the overall quality of the publication as a source of information about treatment choices”) and mean scores of all questions.

	Question 16			Average Score		
Article Name	Reviewer 1	Reviewer 2	Reviewer 3	Reviewer 1	Reviewer 2	Reviewer 3
Recurrent Urinary Tract infections in Adult Women	2	3	2	2.19	3.75	3.69
Increased Daily Water Intake in Premenopausal Women with Recurrent Urinary Tract Infections	3	4	4	2.81	4.06	4.13
Could An Underlying Bladder Infection be Causing your Recurrent UTIs?	2	4	4	2.31	4.13	4.13
Vaginal Bacteria Can Trigger Recurrent UTIs, Study Shows	2	4	5	2.13	4.00	4.00
Vaginal Bacteria Can Trigger Recurrent UTIs, Study Shows	2	4	5	2.13	4.00	4.00
Why Do I Keep Getting UTIs? 5 Causes of Recurrent UTIs	2	5	5	1.31	4.75	4.69
Vaginal bacteria can trigger recurrent UTIs, study shows: Findings help explain UTI link to sexual activity	2	4	4	2.13	4.00	3.88
AUA Releases First Uncomplicated Recurrent UTI Guideline	4	4	4	4.00	4.13	3.75
Recurrent Uncomplicated Urinary Tract Infections in Women: AUA/CUA/SUFU Guideline (2019)	4	5	5	4.00	4.69	4.56
Possible role of L-form switching in recurrent urinary tract infection	3	2	2	2.94	3.44	3.44

**Table 2:** Administered forms regarding recurrent UTI patient questions found ten general categories of concern.

Category	Number of Questions (*n* = 709)
Antibiotics	77
Diet/Lifestyle	38
Hormones	43
Prevention	63
Microbiome	58
Pelvic Pain	65
Sex	59
Symptoms	103
Testing	77
Vaccine	126

## Data Availability

The original contributions presented in this study are included in the article. Further inquiries can be directed to the corresponding author.

## Supplementary Materials

The following supporting information can be downloaded at https://www.mdpi.com/article/10.3390/pathogens13121125/s1.
