# Supplementary material for: Public Interest in Online Information on Recurrent Urinary Tract Infections Is Greatest for Information with the Poorest Publication Quality
Source: Pathogens. 2024 Dec 20;13(12):1125. doi: 10.3390/pathogens13121125 (PMC11679484; doi:10.3390/pathogens13121125)
Supplement: Supplementary file 1 [file pathogens-13-01125-s001.zip › pathogens-3258938-supplementary.pdf]

	Question
1	Are the aims clear?
2	Does it achieve its aims?
3	Is it relevant?
4	Is it clear what sources of information were used to compile the publication (other than the author or producer)?
5	Is it clear when the information used or reported in the publication was produced?
6	Is it balanced and unbiased?
7	Does it provide details of additional sources of support and information?
8	Does it refer to areas of uncertainty?
9	Does it describe how each treatment works?
10	Does it describe the benefits of each treatment?
11	Does it describe the risks of each treatment?
12	Does it describe what would happen if no treatment is used?
13	Does it describe how the treatment choices affect overall quality of life?
14	Is it clear that there may be more than one possible treatment choice?
15	Does it provide support for shared decision-making?
16	Based on the answers to all of the above questions, rate the overall quality of the publication as a source of information about treatment choices.
